# Preparation of Lithium–Cesium Co-Doped Tungsten Oxide by Low-Temperature Hydrothermal Method

**DOI:** 10.3390/nano15211616

**Published:** 2025-10-23

**Authors:** Yue Liu, Xinyu Song, Liying Wen, Yan Luo, Zhiwang Sun, Shifeng Wang

**Affiliations:** Key Laboratory of Plateau Oxygen and Living Environment of Xizang Autonomous Region, College of Science, Xizang University, Lhasa 850000, China; liuyue@stu.utibet.edu.cn (Y.L.); songxinyu@stu.utibet.edu.cn (X.S.); wenliying@stu.utibet.edu.cn (L.W.); luoyan@stu.utibet.edu.cn (Y.L.); szh@stu.utibet.edu.cn (Z.S.)

**Keywords:** tungsten bronze, low-temperature synthesis, plasmonic materials, smart windows, energy efficiency

## Abstract

Buildings consume 40% of global energy, over half of which is used for cooling and heating. Tungsten bronze (M_x_WO_3_) holds promise for smart windows due to its ability to block near-infrared (NIR) heat radiation while maintaining visible light transmittance. However, conventional high-temperature synthesis is energy intensive. Here, we develop a low-temperature hydrothermal method (170 °C) to prepare Li and Cs co-doped tungsten oxide using WCl_6_, LiF, and CsOH·H_2_O as precursors, with acetic acid as a crystallographic modulator. The material exhibits a hexagonal structure (P6_3_/mcm) and Li^+^-induced lattice expansion (0.34 nm spacing). Combined XPS and ICP-OES analyses confirm the chemical composition as Cs_0.31_Li_0.09_WO_3_ and reveal a positive correlation between the W^5+^ content (15.76%) and oxygen vacancy concentration, which is identified as the key factor enhancing the NIR absorption. The material demonstrates excellent visible light transmission and NIR shielding properties. Our work provides a more energy-efficient and sustainable pathway for the production of smart window materials.

## 1. Introduction

The emission of greenhouse gases, such as carbon dioxide and methane, is contributing to global warming and causing various environmental problems. Energy-saving glass with NIR shielding performance can efficiently insulate the heat that primarily originates from the NIR region of sunlight, and reduce the indoor or car temperatures in summer. It can lower the electricity consumption of indoor space-cooling and lighting equipment, thus promoting energy-saving [1,2,3]. From a materials science perspective, one of the keys to achieving this functionality (i.e., high visible light transmission and selective near-infrared shielding) lies in transparent conductive oxides (TCOs). Traditional TCOs, such as indium-doped tin oxide (ITO), are widely used due to their excellent conductivity and transparency. However, their high cost and the scarcity of indium have prompted the exploration of alternative materials. In this context, a range of low-cost, elementally abundant alternative TCO systems have been developed, such as aluminum-doped zinc oxide (ZnO: Al, AZO) and niobium-doped titanium dioxide (TiO_2_: Nb). These materials introduce free charge carriers into wide-bandgap semiconductors through doping, thereby enabling the regulation of near-infrared light via plasmonic resonance effects, making them important members of the transparent thermal insulation material family [4,5]. Currently, various types of transparent insulating materials have been developed, including indium-doped tin oxide (ITO), antimony-doped tin oxide (ATO), non-stoichiometric tungsten oxide (WO_3−X_), gold nanoparticles (n-Au), silver nanoparticles (n-Ag), lanthanum hexaboride nanoparticles (LaB_6_), and hexagonal tungsten bronzes (M_x_WO_3_, where M = Cs^+^, Rb^+^, K^+^, Na^+^, NH_4_^+^, as well as H^+^, which is pivotal for the gasochromic effect) [6,7,8,9,10]. The non-stoichiometric tungsten oxides (WO_3−x_), in which the parameter x of the chemical formula normally ranges from 0 to 1 and represents the degree of oxygen deficiency, have proved superior to WO_3_ for various properties, due to the self-doping effect introduced by the oxygen vacancies in the materials [11,12]. Among these materials, tungsten bronzes have attracted increasing attention due to their relatively low cost and excellent near-infrared (NIR) shielding performance [13,14]. It is noteworthy that the foundational principle of modulating the optical properties of WO_3_ by ion insertion is shared with the well-established electrochromic phenomenon, which was introduced into smart window applications by C. G. Granqvist several decades ago [15,16]. In addition to electrochromism, WO_3_ also possesses an outstanding gasochromic effect. Prof. L. M. Thomas and his group pioneered the work on the gasochromic effect in WO_3_ thin films and also made comparative investigations on the gasochromic and electrochromic effect in the WO_3_ thin films [17,18,19]. To improve the optical property and boost the light modulation of WO_3_ thin films, cation doping was applied, such as Cs^+^ insertion in the WO_3_ lattice, forming a Cs_x_WO_3_ structure. Prof. Li and his team found that the Cs_0.3_WO_3_ is formed through a dissolution–recombination route during the nucleation and growth process [20]. Cs^+^ doping into the WO_3_ crystal could effectively adjust the density of free carriers, thereby enhancing the small polariton absorption ability and the local surface plasmon resonance effect [21]. The continued increase in greenhouse gases is contributing to a warming global climate. According to global average surface temperature measurements, 2021 was one of the six hottest years on record, with lake surface temperatures reaching their highest levels on record and the number of warm days on land reaching an all-time high. Increased carbon emissions from human activities have become a major driver of global climate change, with growth in carbon emissions coming mainly from industry, transportation and energy supply, while residential and commercial buildings, among others, also contribute significant amounts of carbon dioxide, methane and other greenhouse gases [22]; air conditioning and heating systems account for more than 50% of total building energy consumption [23]. With the introduction of China’s “carbon peak and carbon neutral” strategy, controlling carbon emissions has become a key issue in China’s low-carbon transformation and development. Transparent insulation is destined to play an important role in slowing down carbon emissions. It plays a crucial role in reducing the use of indoor air conditioning and vehicle air conditioning.

Researchers have developed various materials that can be utilized as transparent thermal insulation coatings. These materials include conventional infrared shielding materials such as low-emissivity (low-E) coatings [24,25], vanadium dioxide (VO_2_) [26], ITO [27,28], and modified zinc oxide (ZnO) [29,30]. However, these materials possess inherent limitations that hinder their broader applications. The core component of low-E coatings is the low-emissivity Ag layer. To ensure the stability of the Ag layer during operation, a complex multilayer vacuum structure must be designed, resulting in high manufacturing costs [24,25]. VO_2_, one of the most widely studied thermochromic materials, holds significant promise for smart window applications due to its unique reversible metal-insulator transition (MIT). Nevertheless, its relatively high phase transition temperature (Tc = 68 °C), which exceeds room temperature, limited solar modulation ability, and poor oxidation resistance restrict its practical implementation [26]. Both ITO and modified ZnO exhibit excellent infrared shielding properties as transparent insulating materials [27,28]. Recently, hafnium-doped ZnO coatings have been investigated for constructing windows with self-cleaning effects and controllable solar transmittance [29,30]. However, the strong shielding range of ITO and modified ZnO is primarily concentrated in the 1500–2500 nm region, while their near-infrared (NIR) transmittance remains high in the 780–1500 nm range, leading to suboptimal NIR shielding efficiency [27]. Moreover, indium, a critical component of ITO, is a rare and expensive metal, further limiting its large-scale commercial viability.

Tungsten bronze materials (M_x_WO_3_, M = Li [31], Na [32], Cs [33], etc.) have garnered considerable research interest owing to their promising applications in energy-saving windows. The NIR shielding property of tungsten bronze powder is attributed to the localized surface plasmon resonance (LSPR) and small polaron transfer [34,35]. It is noteworthy that the fundamental understanding of the polaron absorption mechanism in reduced tungsten oxides dates back to seminal early studies. Saenger’s research group discovered that two polaron modes exist in the electron transitions between W^4+^ and W^5+^ and between W^5+^ and W^6+^ for tungsten ion sites [36]. Additionally, the direct spectroscopic evidence for these mixed valence states was determined through XPS investigations of the tungsten oxide films [37]. Among the many transparent insulation materials, alkali metal-doped tungsten bronze material is widely noticed for its excellent electrical and color rendering properties. Its main principle of action is the selective transmission of the visible wavelengths of sunlight and the selective absorption of the near-infrared wavelengths that can raise the ambient temperature, thus avoiding ambient heating and reducing the use of air-conditioning, thereby slowing down global warming and reducing carbon emissions. Bo xu et al. used ammonium meta-tungstate as the tungsten source and lithium chloride as the lithium source to obtain lithium–cesium co-doped tungsten-bronze by hydrothermal reaction at 240 °C for 24 h, and enhanced the spectral tunability of LSPR and small polaron transfer [38]. Hao yuan et al. mixed CsNO_3_, Li_2_CO_3_, and WO_3_ with deionized water, with M/W (M = Li + Cs) and Li/Cs molar ratios of 0.35 and 0.2:0.8, respectively. Then, the well-mixed raw materials were annealed in a high-temperature tube furnace at 650 °C under a H_2_ atmosphere for 30 min and naturally cooled to room temperature to obtain LimCsnWO_3_ nanoparticles. It has been confirmed that the LimCsnWO_3_ nanoparticles with Li/Cs = 0.2:0.8 prepared at 650 °C have the best near-infrared shielding performance, with the maximum visible light transmittance reaching 75.18%, and the NIR shielding rate attaining 97.65% [23].

Our study presents a breakthrough low-temperature (170 °C) hydrothermal method for synthesizing phase-pure Cs_x_Li_y_WO_3_ nanocrystals. By employing acetic acid as a crystallographic modulator, we successfully achieved co-doping of Li^+^ and Cs^+^, and confirmed the chemical composition and W^5+^/W^6+^ mixed-valence states through characterization techniques such as XPS and ICP-OES. Notably, our O 1s XPS analysis established a direct correlation between the material’s performance and its oxygen vacancy concentration, providing experimental evidence for understanding its excellent near-infrared shielding properties. This synthesis strategy consumes significantly less energy compared to conventional high-temperature methods. Our work not only provides a scalable pathway for low-carbon material fabrication but also offers new perspectives for designing next-generation smart window materials by elucidating the relationship between oxygen vacancy concentration, valence state, and performance in co-doped tungsten bronzes.

## 2. Materials and Methods

### 2.1. Materials

The following reagents are of analytical grade and utilized as received without further treatment. Tungsten chloride (WCl_6_, 99%), Cesium hydroxide monohydrate (CsOH·H_2_O, AR, 95%), Lithium fluoride (LiF, AR, 99%), acetic acid (CH_3_COOH, AR, 99.5%), and anhydrous ethanol (C_2_H_5_OH) were purchased from Shanghai Aladdin Biochemical Technology Co., Ltd. (Shanghai, China).

### 2.2. Synthesis of LimCs_0.5_WO_3_ Nanoparticles

**Figure 1:**
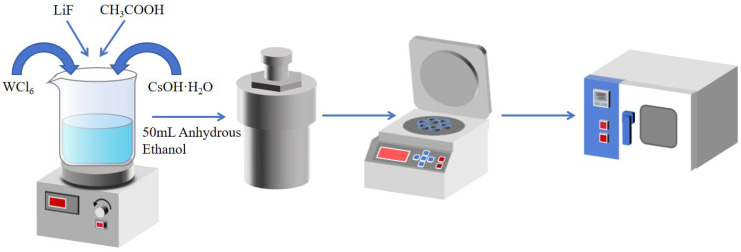
Schematic workflow for the synthesis of Cs-Li co-doped tungsten bronze nanoparticles.

The synthesis of LimCs_0.3_WO_3_ was carried out according to the following procedure. 1 mmol of WCl_6_ was dissolved in 50 ml of ethyl alcohol, followed by the addition and complete dissolution of 0.5 mmol CsOH·H_2_O. The crystalline water in CsOH·H_2_O promotes its dissolution in ethanol, enabling molecular-level dispersion of the cesium precursor. This operation was repeated five times to produce five identical mixtures. After that, LiF with different molar masses (0.5 mmol, 1.0 mmol, 1.5 mmol, 2.0 mmol, and 2.5 mmol) was added to the five mixtures in turn. Despite its extremely low solubility in ethanol, LiF undergoes gradual etching under the following acidic hydrothermal conditions, thereby releasing Li^+^ ions. Then 12.5 mL of acetic acid was introduced to each mixture, followed by additional stirring. In this system, acetic acid functions dually as a crystallization moderator and reaction catalyst. Its role involves both proton-assisted acceleration of WCl_6_ hydrolysis/condensation and carboxyl-directed coordination that modulates crystallization kinetics, ultimately promoting the formation of a highly crystalline hexagonal nanostructure. Five mixtures were then transferred into five 100 mL Teflon-lined stainless-steel autoclaves (Shanghai Xiniu Leibo Instrument Co., Ltd., Shanghai, China) and heated at 220 °C for 24 h. After cooling to room temperature, the samples were washed with ethyl alcohol and dried in a vacuum oven (Shanghai Jinghong Experimental Equipment Co., Ltd., Shanghai, China) at 60 °C for 12 h (As shown in Figure 1). The obtained materials were labeled Li_0.5_Cs_0.5_WO_3_, Li_1.0_Cs_0.5_WO_3_, Li_1.5_Cs_0.5_WO_3_, Li_2.0_Cs_0.5_WO_3_, and Li_2.5_Cs_0.5_WO_3_ (LimCs_0.5_WO_3_). Li_1.0_Cs_0.5_WO_3_ was synthesized at different temperatures (160 °C, 170 °C, 180 °C, 190 °C, and 200 °C).

### 2.3. Characterization

XRD patterns of the samples were recorded on a Bruker D8 ADVANCE X-ray diffractometer (Bruker AXS GmbH, Billerica, MA, USA) using filtered Cu-Kα radiation (λ = 1.5406 Å), generated at 40 kV and 40 mA. Scans for 2θ values were recorded at 6° min-1 between 10° and 90°. SEM images were obtained on a Hitachi (SU8020) scanning electron microscope (Hitachi Ltd., Tokyo, Japan). Energy dispersive spectroscopy (EDS, Hitachi Ltd., Tokyo, Japan) was employed for determining the elemental composition of the materials. TEM micrographs were taken on a JEOL (JEM-2100 F) transmission electron microscope (JEOL Ltd., Tokyo, Japan) operating at 200 kV. X-ray photoelectron spectra (XPS) were acquired with a Thermo ESCALAB 250 Xi spectrometer (Waltham, MA, USA). UV−visible−NIR absorption spectra were measured using a Shimadzu (UV-3600, 300–2500 nm) spectrophotometer (Shimadzu Corporation, Kyoto, Japan).

## 3. Results

**Figure 2:**
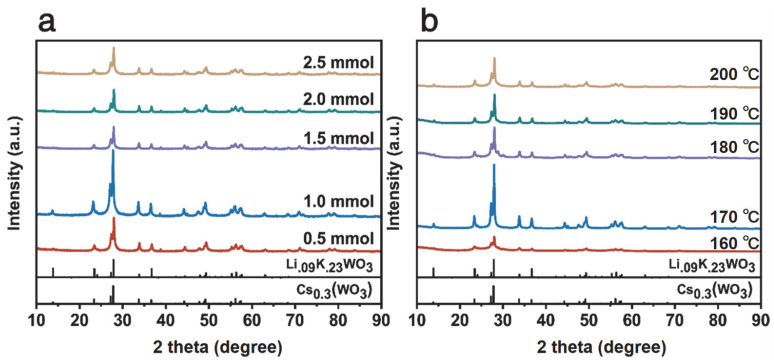
XRD patterns of LimCs_0.5_WO_3_: (**a**) different Li contents; (**b**) Cs_0.31_Li_0.09_WO_3_ prepared at different temperatures.

Figure 2a displays the X-ray diffraction (XRD) patterns of the Cs_0.5_Li_y_WO_3_ series. All samples exhibit sharp and intense diffraction peaks, indicating a highly crystalline hexagonal tungsten bronze (HTB) structure (space group: P6_3_/mcm). The diffraction peaks observed at 23.35°, 27.27°, 27.97°, 33.84°, and 36.69° can be indexed to the (002), (102), (200), (112), and (202) planes of the HTB structure, respectively. This indexing was performed based on reference patterns for the isostructural hexagonal tungsten bronze compounds Cs_0.3_WO_3_ (PDF#97-007-2618) and Li_0.09_K_0.23_WO_3_ (PDF#97-006-5729) [39,40]. Notably, a systematic shift in diffraction peak positions is observed with variations in the Li precursor content. This phenomenon provides strong evidence for the introduction of microstrain within the crystal lattice by successful Li^+^ doping. Such peak shifts are an effective method for analyzing crystal strain [41]. Among the series, the sample with the nominal composition Cs_0.5_Li_1.0_WO_3_ demonstrates the highest diffraction peak intensity, signifying its optimal crystallinity. Figure 2b shows the XRD patterns of Cs_0.31_Li_0.09_WO_3_ samples synthesized at different temperatures (160–200 °C). The sample synthesized at 170 °C exhibits significantly higher intensity for the (200) peak at approximately 27.97° compared to other temperatures, indicating the most complete crystallization at this temperature. Furthermore, a discernible shift in this sample’s (200) peak to lower angles is observed when compared to the reference patterns. According to Bragg’s law, this directly corresponds to an increase in interplanar spacing, i.e., lattice expansion, thereby directly confirming the presence of tensile strain induced by Li^+^ doping [1,42]. This lattice strain, acting synergistically with the W^5+^/W^6+^ mixed valence states revealed by subsequent XPS analysis, is considered a key structural feature significantly enhancing the material’s near-infrared shielding performance [33,34].

ICP-OES was applied to accurately characterize the chemical composition of the prepared samples, as listed in Table 1, which presents the elemental weight percentages and the corresponding atomic ratios of Cs/W and Li/W. It is obvious that the detection of the ratio of Cs to W in the sample remains relatively stable, ranging between 0.29 and 0.34, which aligns with the capacity of the hexagonal tungsten bronze structure to accommodate large-radius alkali metal ions (the theoretical value of x = 0.33). By contrast, the Li content (y value) increases gradually from 0.30 to 1.07 with the increasing Li precursor dosage, demonstrating effective control over Li incorporation during the preparation process. The total alkali ion content (x + y) is a critical parameter for identifying the crystal phases of the synthesized samples. The (x + y) values for the samples with Li doping below 1 mmol range from 0.30 to 0.40, falling within the physical limit for the hexagonal tungsten bronzes (x ≈ 0.3–0.5). In contrast, the (x + y) values for samples of Li doping exceeding 1.5 mmol are as high as 0.83 and 1.07, significantly breaking the limit of 0.5. These results demonstrate a deviation from phase purity under high Li loading, where excessive Li incorporation probably leads to the formation of some secondary crystalline phases, such as lithium oxides or lithium tungstates.

**Figure 3:**
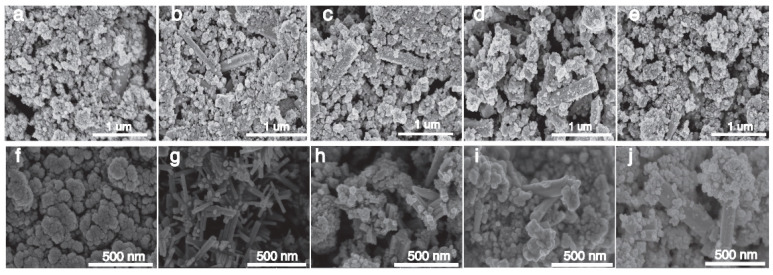
SEM images of Cs_0.5_Li_y_WO_3_: (**a**–**e**) different Li precursor amounts (0.5–2.5 mmol); (**f**–**j**) Cs_0.31_Li_0.09_WO_3_ synthesized at different temperatures (160–200 °C) and elemental compositions.

Figure 3a–e shows the SEM images of Cs_0.5_Li_y_WO_3_ doped with 0.5–2.5 mmol LiF, and the generation of strips can be clearly seen, especially the 1.0 mmol LiF doped sample, which generates strips with a smooth surface and no impurity particles attached. It shows that the reaction is more complete when the content of LiF is 1.0 mmol, which is more suitable for the generation of lithium–cesium co-doped tungsten oxide. Figure 3a–f shows the SEM images of the Cs_0.31_Li_0.09_WO_3_ samples at 160–200 °C, and it can be seen that a large number of strips are formed at 170 °C with interlaced growth, and the surface is smooth with no impurity particles attached, which indicates that the reaction of the Cs_0.31_Li_0.09_WO_3_ is more complete under the condition of 170 °C, and the product is more densely populated, which offers a possibility of a strong NIR shielding properties.

**Figure 4:**
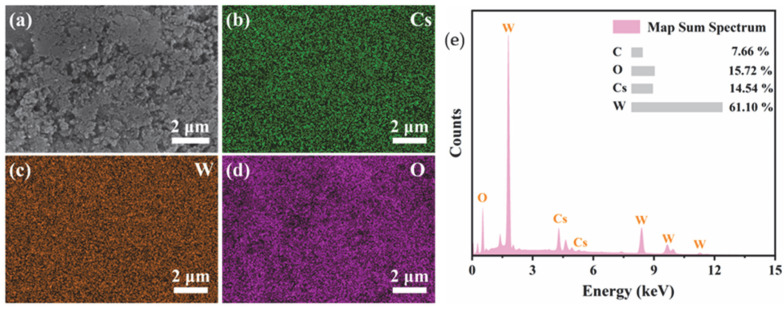
SEM-EDS elemental mapping images of the Cs_x_Li_y_WO_3_ sample. (**a**) Secondary electron image, (**b**) Cs map, (**c**) W map, (**d**) O map, and (**e**) elemental composition.

Figure 4 shows the SEM-EDS mapping and the corresponding elemental composition. It is seen that the Cs, W, and O elements are uniformly distributed on the feature in the selected mapping area. It is worth noting that the distribution of Cs perfectly coincides with that of W and O, indicating that Cs^+^ ions have been successfully and uniformly incorporated into the crystal tunnels of the hexagonal tungsten oxide. The characteristic X-ray peaks of Cs, W, O, and C are labeled in Figure 4e. The atomic percentages (at%) provided in the inset table yield a Cs/W atomic ratio of approximately 0.24.

**Figure 5:**
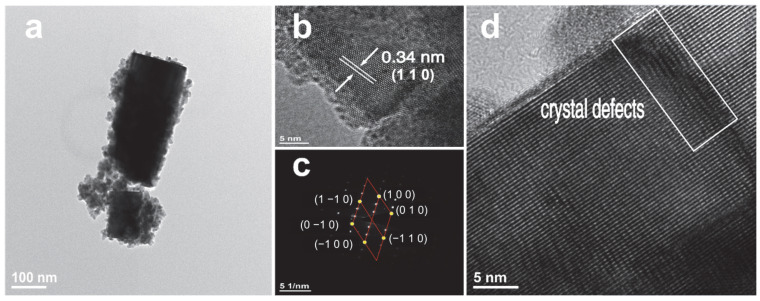
(**a**–**d**) TEM images of Cs_0.31_Li_0.09_WO_3_ sample.

Figure 5 illustrates the TEM images of the prepared Cs_0.31_Li_0.09_WO_3_ sample. Figure 5a reveals the typical rod-like morphology with a diameter of approximately 150 nm. The HRTEM image in Figure 5b presents distinct lattice fringes with a measured spacing of 0.34 nm, which can be assigned to the (110) planes of the hexagonal Cs_x_Li_y_WO_3_ structure. Normally, the interplanar spacing of (110) planes for Cs_0.33_WO_3_ is 0.37 nm. The slightly smaller value for the Cs_x_Li_y_WO_3_ is probably ascribed to the lattice strain induced by the incorporation of Li ions. The corresponding selected-area electron diffraction (SAED) pattern is shown in Figure 5c. The sharp diffraction spots with clear sixfold symmetry confirm the hexagonal crystalline nature of the fabricated Cs_0.31_Li_0.09_WO_3_. Furthermore, the presence of crystal defects, as indicated by the rectangular box in Figure 5d, is consistent with the microscopic strain fields potentially introduced by Li^+^ incorporation into the host lattice.

**Figure 6:**
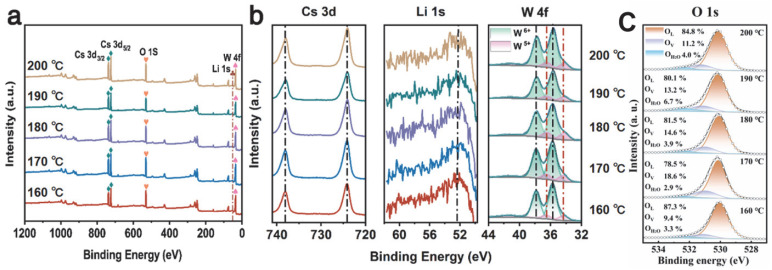
XPS of Cs_0.5_Li_1.0_WO_3_ powders synthesized at different temperatures: (**a**) survey, (**b**) Cs 3d, Li 1s, W 4f and (**c**) O 1s spectra.

The surface chemical composition of the 160–200 °C Cs_0.31_Li_0.09_WO_3_ samples was determined by XPS characterization in Figure 6a. The survey spectra and high-resolution Cs 3d, Li 1s, W 4f XPS of 160–200 °C Cs_0.31_Li_0.09_WO_3_ samples are shown in Figure 6. The peaks at 724.21 eV and 738.07 eV correspond with Cs^+^ 3d_5/2_ and Cs^+^ 3d_3/2_ orbitals, respectively. The peaks at 52.5 eV can be attributed to the Li 1s orbital, which is in good agreement with the reported XPS results of Li [42]. The high-resolution XPS of W 4f can be fitted to two spin–orbit dual states of W 4f_7/2_ and W 4f_5/2_. W^6+^ and W^5+^ are responsible for the spin–orbit peaks at 35.66 eV/37.85 eV and 34.33 eV/36.54 eV, respectively. The content of W^5+^ in 160 °C Li_1.0_Cs_0.5_WO_3_ sample is the least, accounting for 12.84% of the total W atoms. The content of W^5+^ increases and then decreases with the rise in temperature, and the highest relative content of 15.76% is found at the temperature of 170 °C. It is due to increased lithium doping, and the afforded electron plays the role of the reducing agent for the W atoms [43]. This is also responsible for the enhanced NIR shielding performance [32]. The relative content of W^5+^ decreases from 15.55% at 180 °C to 14.31% and finally to 13.91% at 200 °C. It is consistent with the TEM results. Figure 6c presents the high-resolution XPS spectrum of O 1s, in which the different components originating from lattice oxygen (O_L_, at 530.4 eV), oxygen vacancies (O_V_, at 531.5 eV), and surface-adsorbed water/hydroxyl groups (O_H2O_, at 532.8 eV) are deconvoluted to investigate the role of oxygen vacancies in-depth. Quantitative analysis of O_V_ reveals that its concentration increases from 9.4% at 160 °C to a peak of 18.6% at 170 °C, and then decreases gradually to 13.2% with the increasing preparation temperature. This trend shows a very strong positive correlation with the W^5+^ fraction from W 4f XPS, which suggests that the W^5+^ states originate directly from the oxygen vacancies, not from the Li doping. Each oxygen vacancy produces two electrons, locally reducing two W^6+^ to W^5+^ (or one to W^4+^) for charge balance. Thus, the W^5+^ maximum at 170 °C is intrinsically governed by the peak in oxygen vacancy concentration at this optimal temperature, confirming an oxygen vacancy-dominated charge compensation mechanism.

**Figure 7:**
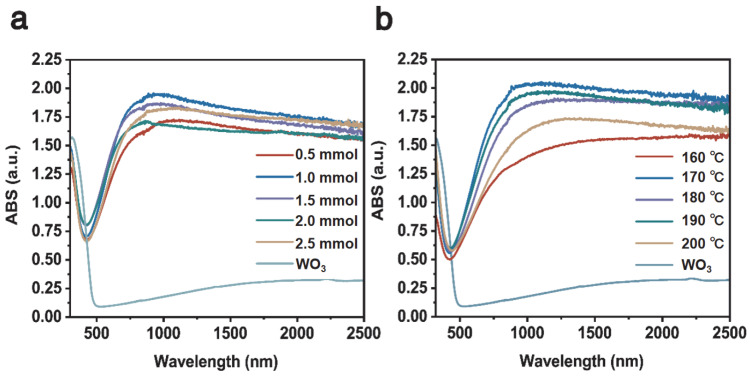
UV–Visible–NIR absorption spectrum of (**a**) the Cs_0.5_Li_y_WO_3_ with varying Li doping; (**b**) the Cs_0.5_Li_1.0_WO_3_ prepared at varying temperatures of 160–200 °C.

Figure 7a shows the UV–Visible–NIR absorption of Cs_0.31_Li_y_WO_3_ samples with varying Li doping. It is obvious that the sample of Cs_0.31_Li_0.09_WO_3_ possesses a better absorption ability in the NIR wavelength. Figure 7b shows the UV–Visible–NIR absorption of the Cs_0.31_Li_0.09_WO_3_ sample prepared at varying temperatures, in which the sample synthesized at 170 °C exhibits the best absorption property in the wavelength range between 800 nm and 2500 nm while maintaining almost the same level of absorption in the visible range for the doped WO_3_. The results are consistent with the findings of the lithium intercalated tungsten-bronze system [42]. These optical measurements demonstrate that the optimum Cs:Li for synthesizing high-quality Cs_x_Li_y_WO_3_ for the transparent heat insulation applications is 0.5:1.0 and the optimum temperature is 170 °C.

## 4. Conclusions

In this study, Cs_0.31_Li_0.09_WO_3_ material with high NIR shielding performance was successfully synthesized via a simple low-temperature hydrothermal method. Compared to conventional high-temperature synthesis, this approach significantly reduces energy consumption. The effects of LiF doping content and reaction temperature on the product’s morphology, crystal structure, elemental composition, valence state, and optical properties were systematically investigated. The results indicate that the sample synthesized at 170 °C with a Cs/Li molar ratio of 0.5:1.0 possesses the highest crystallinity. O 1s XPS analysis confirmed that this optimal sample has the highest oxygen vacancy concentration, which correlates directly with the peak in W^5+^ content (15.76%) observed in W 4f XPS. This identifies the oxygen vacancy-dominated charge compensation mechanism as the intrinsic reason for the enhanced NIR shielding performance, rather than the effect of Li doping content alone. This optimal sample exhibits excellent overall optical properties. This work not only provides a feasible strategy for the green preparation of high-performance tungsten bronze materials but also deepens the understanding of the relationship between oxygen vacancies and optical properties in co-doped systems. Future work will focus on evaluating the long-term stability of this material under simulated practical conditions and fabricating it into smart window coatings for actual application testing to assess its real-world energy-saving performance, thereby accelerating its potential for industrial adoption.

## Figures and Tables

**Table 1:** Chemical composition of the as-synthesized samples determined by ICP-OES.

Sample	Determined Composition (Cs_x_Li_y_WO_3_)	Cs	W	Li	x (Cs/W)	y (Li/W)	Total Alkali (x + y)
Cs_0.5_WO_3_	Cs_0.30_Li_0_WO_3_	12.32	57.45	0	0.30	0.00	0.30
Cs_0.5_Li_0.5_WO_3_	Cs_0.29_Li_0.03_WO_3_	12.90	60.97	0.06	0.29	0.03	0.32
Cs_0.5_Li_1.0_WO_3_	Cs_0.31_Li_0.09_WO_3_	13.23	59.11	0.19	0.31	0.09	0.40
Cs_0.5_Li_1.5_WO_3_	Cs_0.33_Li_0.26_WO_3_	12.36	53.44	1.03	0.32	0.51	0.83
Cs_0.5_Li_2.0_WO_3_	Cs_0.35_Li_0.45_WO_3_	14.58	59.20	1.64	0.34	0.73	1.07

## Data Availability

The original contributions presented in this study are included in the article. Further inquiries can be directed to the corresponding author.
